# Endoparasite diversity and liver alterations in *Hoplerythrinus unitaeniatus* and *Cichlasoma bimaculatum* in a quilombola area in Maranhão, Brazil

**DOI:** 10.1590/S1984-29612022022

**Published:** 2022-04-20

**Authors:** Ladilson Rodrigues Silva, Vitorya Mendes da Silva Monteiro, Izabela Alves Paiva, Juliany Silva Mendes, Greiciene dos Santos de Jesus, Marcelo Victor Rodrigues da Silva, Danilo Cutrim Bezerra, Carlos Riedel Porto Carreiro, Larissa Sarmento dos Santos Ribeiro, Viviane Correa Silva Coimbra, Nancyleni Pinto Chaves Bezerra

**Affiliations:** 1 Programa de Pós-graduação Acadêmico em Ecologia e Conservação da Biodiversidade, Centro de Educação, Ciências Exatas e Naturais, Universidade Estadual do Maranhão – UEMA, São Luís, MA, Brasil; 2 Curso de Graduação em Ciências Biológicas, Centro de Educação, Ciências Exatas e Naturais, Universidade Estadual do Maranhão – UEMA, São Luís, MA, Brasil; 3 Curso de Graduação em Engenharia de Pesca, Centro de Ciências Agrárias, Universidade Estadual do Maranhão – UEMA, São Luís, MA, Brasil; 4 Curso de Graduação em Zootecnia, Centro de Ciências Agrárias, Universidade Estadual do Maranhão – UEMA, São Luís, MA, Brasil; 5 Programa de Pós-graduação Profissional em Defesa Sanitária Animal, Centro de Ciências Agrárias, Universidade Estadual do Maranhão – UEMA, São Luís, MA, Brasil

**Keywords:** Native fish, traditional communities, Anisakidae, histology, Peixes nativos, comunidades tradicionais, Anisakidae, histologia

## Abstract

Our aim was to assess endoparasite diversity and liver alterations in *Hoplerythrinus unitaeniatus* (jeju) and *Cichlasoma bimaculatum* (acará preto) in a quilombola area in Maranhão, Brazil. For this, 21 *H. unitaeniatus* and 21 *C. bimaculatum* were caught in a natural environment and transported to a laboratory. After these had been euthanized, endoparasites were collected and identified. Liver alterations were evaluated histological analysis based on the severity of each lesion: stage I, organ functioning not compromised; stage II, more severe lesions that impair normal functioning of the organs; and stage III, very severe and irreversible lesions. Among the fish evaluated, 71.43% *H. unitaeniatus* and 61.90% *C. bimaculatum* were parasitized. *Contracaecum* sp. was found in both species; while acanthocephalans, only in *H. unitaeniatus*. The alterations were vacuolization, nucleus in the cell periphery, deformation of the cell outline, melanomacrophage center, hyperemia, cytoplasmic degeneration and nuclear vacuolization. Through calculating a histological alteration index, it was found that 26.19% of the specimens presented lesions in stage I; 38.09% lesions in stage II and 9.52% lesions in stage III. It was concluded that there is high prevalence of *Contracaecum* sp. and that the liver lesions may be adaptive responses by the fish to endoparasitic infection.

## Resumo

Objetivou-se avaliar a diversidade de endoparasitos e alterações hepáticas em *Hoplerythrinus unitaeniatus* (jeju) e *Cichlasoma bimaculatum* (acará preto) de área quilombola maranhense, Brasil. Assim, 21 *H. unitaeniatus* e 21 *C. bimaculatum* foram capturados de ambiente alagável e transportados vivos ao laboratório. Após a eutanásia, procedeu-se à coleta, identificação de endoparasitos e avaliação de alterações hepáticas por meio de análise histológica, baseada na severidade das lesões: (i) alterações de estágio I, não comprometem o funcionamento dos órgãos; (ii) estágio II, lesões mais severas que prejudicam o funcionamento normal dos órgãos; (iii) estágio III, lesões muito severas e irreversíveis. Dos peixes avaliados, 71,43% *H. unitaeniatus* e 61,90% *C. bimaculatum* estavam parasitados. *Contracaecum* sp. foi encontrado nas duas espécies e acantocéfalos apenas em *H. unitaeniatus*. As alterações hepáticas foram vacuolização, núcleo na periferia das células, deformação do contorno celular, centro de melanomacrófagos, hiperemia, degeneração citoplasmática e vacuolização nuclear. Com o cálculo do índice das alterações, constatou-se que 26,19% dos exemplares apresentaram alterações de estágio I; 38,09% de estágio II e 9,52% de estágio III com a constatação de larvas de *Contracaecum* sp. encistadas no fígado. Conclui-se que existe alta prevalência de *Contracaecum* sp., e lesões hepáticas podem ser respostas adaptativas dos peixes à infecção endoparasitária.

## Introduction

The factors that control the diversity of parasitic species and levels of infection in fish are often ecological (Moreira et al., 2009; Tavares-Dias et al., 2014; Alcântara & Tavares-Dias, 2015). In impacted environments, histological biomarkers can be used as a diagnostic tool for determining the health of fish populations, which reflects the condition of an aquatic ecosystem as a whole (Pereira et al., 2014). Among fish, histological studies are directed to different organs, especially that are responsible for metabolism, such as the liver, which may undergo structural and metabolic alterations through exposure to pollutants, contaminated food, toxins, microorganisms and parasites (Rocha et al., 2010).

Imbalances between the environment, the host and the etiological agent can result in occurrences of diseases, including parasitic diseases (Takemoto et al., 2009; Alves et al., 2019). The characteristics inherent to the aquatic environment facilitate propagation, reproduction and complementation of the life cycle of parasitic agents (Pavanelli et al., 2008).

Knowledge of parasites in natural fish populations enables evaluation of their impact on their hosts, since many parasites can influence the structure, size, diet, growth rate and reproduction of natural fish populations (Moreira et al., 2009; Takemoto et al., 2009; Tavares-Dias et al., 2014), together with the quality and acceptance of parasitized fish in the consumer market (Benigno et al., 2014). Furthermore, studies on parasitic infections in fish populations generate important information about the parasite-host relationship (Alcântara & Tavares-Dias, 2015).

In the jeju (*Hoplerythrinus unitaeniatus*), infestations and parasitic infections caused by crustacean species (Leal et al., 2010), helminths (Martins et al., 2005; Benigno et al., 2012; Alcântara & Tavares-Dias, 2015), acanthocephalans (Takemoto et al., 2009) and protozoa (Alcântara & Tavares-Dias, 2015) have been reported. In the black acará (*Cichlasoma bimaculatum*), Tavares-Dias et al. (2017) reported infections caused by protozoa and different helminths.

However, there is no information on the endoparasite composition of these two species in the western lowlands of Maranhão, despite the importance of this region. These lowlands form a hydrographic system composed of rivers, lakes and floodplains that has a territorial extent of 1,775,035.9 hectares (ha). Thus, this region has the status of the largest lacustrine basin in the northeastern region of Brazil (Pereira et al., 2016). These two fish species form the food resources for many traditional families in the state of Maranhão.

Despite the importance of artisanal fishing for people in the western lowland area of Maranhão, this activity has been shown to be strongly impacted by anthropic and ecological changes. These have converged to losses of fish species, year after year. Hence, the aim of this study was to assess the endoparasite diversity and liveralterations in *Hoplerythrinus unitaeniatus* (jeju) and *Cichlasoma bimaculatum* (acará preto) in a quilombola area in Maranhão, Brazil.

## Material and Methods

### Legal authorization

This study was approved by the Ethics Committee for Animal Experimentation (CEEA) of the Universidade Estadual do Maranhão (UEMA), under protocol nos. 08/2021. It also complied with Federal Council of Veterinary Medicine (CFMV) resolution nos. 879/2008 and 1000/2012 and with federal law no. 11794/2008, which deal with ethical procedures in animal experimentation.

### Study area

The fish used in this study were caught in a flooded field in the *quilombo* of Ponta Bonita, a traditional community belonging to the municipality of Anajatuba, Maranhão. This municipality lies within the Baixada Maranhense (Maranhão lowlands) region, which is located at the geographical coordinates of latitude 03º15'50” S and longitude 44º37'12” W. The dominant climatic type in this region is AW (humid tropical climate with a dry winter season), according to the climatic classification of Koeppen (1948).

### Fish catch

The fish were caught in fields that are naturally flooded in the rainy season (February 2021) and in the dry season (August 2021). A total of 42 adult specimens (both males and females) were caught: 21 individuals of the species *H. unitaeniatus* (18.7 ± 2.5 cm and 76.65 ± 25.7 g) and 21 of *C. bimaculatum* (12.45 ± 0.77 cm and 44.32 ± 9.80 g). An actively operated mesh net of size 4 (20 mm) was used for catching the fish (as a dragnet/enclosure), and also a cast net of mesh size 4 (20 mm) was used. The specimens thus caught were packed alive for transportation, in a 100-liter Styrofoam box with water from the capture environment along with oxygenation equipment, to the UEMA Aquatic Resource Reproduction Laboratory (LARAQUA). There, they were placed in a tank with water and constant oxygenation for a period of 12 hours until further analysis at the parasitology laboratory of UEMA’s Agrarian Sciences Center (CCA).

### Collection and identification of parasites

Nematodes were collected by means of dissection of the muscles, liver, surface and interior of the digestive tube and fat. They were then placed in a 0.65% saline solution. This was shaken to remove impurities and remains of the fish musculature. The nematodes were then fixed in AFA (alcohol - formalin - acetic acid) at 65 °C, for 48 hours. Following this, they were preserved in 70% alcohol. To identify the nematodes, they were subjected to a dehydration process in an ascending series of alcohols (70%, 80%, 90%) for approximately three hours and were then left overnight in pure alcohol. On the next day, they were clarified in creosote for a minimum of three hours. They were then mounted between slides and coverslips, in a semipermanent histological preparation, for observation of their internal structures and identification (Eiras et al., 2006; Jerônimo et al., 2012).

Acanthocephalans were carefully collected from the digestive tube with the aid of narrow-pointed tweezers. They were then cleaned in physiological saline solution to remove any adherent mucus, placed on Petri dishes together with distilled water and left to die in a refrigerator. Following this, they were placed in 70% alcohol for approximately eight hours. They were then subjected to a dehydration process in an ascending series of alcohols (75%, 85%, 90%, 96% and pure alcohol) for approximately 10 horas. After this, they were transferred to a drop of cedar oil that had been placed on a slide, and then covered with a coverslip. To avoid evaporation of the cedar oil and enable observation of the internal organs and identification of the specimens, a drop of balsam was added around the coverslip (Eiras et al., 2006).

To identify the endoparasites, the methodologies proposed by Moravec et al. (2002), Pardo et al. (2008) and Jaramillo-Colorado et al. (2015) were followed. The prevalence and mean intensity of endoparasites in the fish were determined as described by Bush et al. (1997) and Jerônimo et al. (2012).

### Histological analyses

The histological analyses were performed in the microscopy laboratory that forms part of the Multiuser Postgraduate Laboratory of UEMA. Firstly, the liver fragments were sliced up, so as to reduce their dimensions to a thickness of 3 to 5 mm, in order to facilitate penetration and diffusion of the reagents that would be used in subsequent stages of the histological processing.

Stages of dehydration, diaphanization, embedding in paraffin and microtomy were then followed, as described by Caputo et al. (2010). Transverse sections of approximate thickness 5 μm were cut and stained with hematoxylin and eosin. The slides thus prepared were read under an optical microscope, at magnifications of 10x and 40x, and the lesions found were photomicrographed under a Zeiss Axioscope photomicroscope.

The hepatic histological alterations were evaluated semiquantitatively, by calculating the histological alteration index (HAI), adapted from Poleksic & Mitrovic-Tutundzic (1994). This was based on the severity of each lesion, as follows: (i) stage I alterations – organ functioning not compromised; (ii) stage II - more severe lesions that impair the normal functioning of the organs; and (iii) stage III - very severe and irreversible lesions.

For each fish, the HAI value was calculated using the formula: HAI = 1×Σ *I* + 10×Σ *II* + 100×Σ *III*, where I, II and III correspond respectively to the numbers of alterations in the tissue. The HAI value was defined in five categories established by Poleksic & Mitrovic-Tutundzic (1994): 0-10 = normal tissue functioning; 11-20 = mild to moderate tissue damage; 21-50 = moderate to severe tissue modification; 51-100 = severe tissue modification; and greater than 100 = irreparable tissue damage.

### Statistical analysis

To verify whether there were any significant differences in endoparasite prevalence at the 5% level, between the times evaluated according to species and between the species sampled, the Tukey test was performed using the GraphPad InStat 3.1 free software.

## Results and Discussion

Among the fish evaluated, 71.43% (n = 15/21) of the *H. unitaeniatus* specimens and 61.90% (13/21) of the *C. bimaculatum* specimens were parasitized (Table 1). This prevalence was lower than what was obtained by Rodrigues et al. (2017), who found that 91.40% of their specimens were infected, in an evaluation on 70 specimens of *Hoplias malabaricus* (traíra) in the municipality of São Bento, Maranhão. This difference may have been related to the following variables: species evaluated, seasonal period, capture site, presence in greater or lesser number of reservoirs and intermediate hosts (Moravec et al., 2002).

**Table 1:** Prevalence and mean intensity of endoparasites in *Hoplerythrinus unitaeniatus* and *Cichlasoma bimaculatum* from a *quilombola* area in Maranhão, Brazil.

**Periods**	**N**	**IF**	**TNP**	*Hoplerythrinus unitaeniatus*			
				*Contracaecum* sp.		Acanthocephalans	
				**P**	**MI**	**P**	**MI**
Rainy	3	2	4	66.67	2.00	0.00	0.00
Dry	18	13	61	72.22	3.54	16.67	5.00
**Total**	21	15	65	71.43	3.84	14.28	5.00
**Periods**	**N**	**IF**	**TNP**	*Cichlasoma bimaculatum*			
				*Contracaecum* sp.		Acanthocephalans	
				**P**	**MI**	**P**	**MI**
Rainy	18	10	23	55.55	2.3	0.00	0.00
Dry	3	3	9	100.00	3.00	0.00	0.00
**Total**	21	13	32	61.90	1.52	0.00	0.00

Where: N= amount of fish; IF = infected fish; TNP = total number of parasites collected; P = prevalence; MI = mean intensity.

There was no statistically significant difference (p > 0.05) in the prevalences of endoparasites between the times evaluated according to species, or between the species sampled. However, it should be noted that parasitism in fish is a challenge, regardless of the period of the year, due to the complexity of the factors involved, i.e. those inherent to the hosts (age, immune status, intercurrent diseases, etc.), parasitic agents (species and pathogenicity) or even the environment (climate, local hygiene, biosecurity, reservoirs, etc.) (Fujimoto et al., 2019).

Regarding the mean intensity (MI), it was observed that the nematode *Contracaecum* sp. of the family Anisakidae was predominant in both species (Table 1). This result shows that there was high prevalence of endoparasites. Similar results were found by Martins et al. (2005), Alcântara & Tavares-Dias (2015) and Tavares-Dias et al. (2017) through evaluating specimens of *H. unitaeniatus* and *C. bimaculatum*.

**Figures 1:**
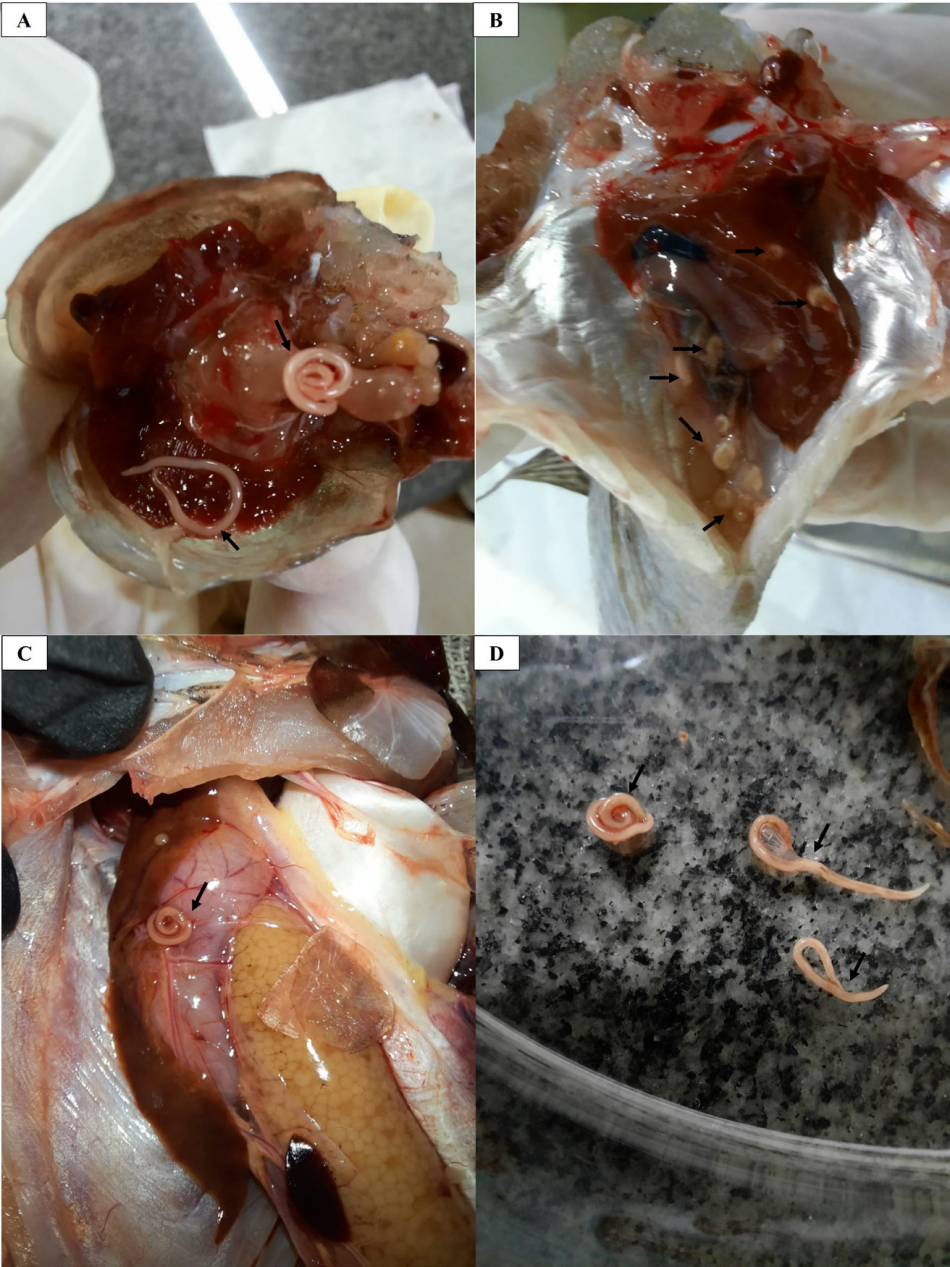
*Contracaecum* sp. nematodes in the native fish *Hoplerythrinus unitaeniatus* (jeju) and *Cichlasoma bimaculatum* (acará preto), caught in a quilombola community in Maranhão: (A) free nematodes in the celomatic cavity; (B) larvae in the celomatic cavity; (C) nematode adhering to the serosa of the stomach; (D) helminths recovered, of different sizes. **Source:** Authors' files.

*Contracaecum* sp. of different sizes and at larval stage L3 was found in organs of the celom cavity, encapsulated on organs in this cavity (liver and mesentery), and in the serosa of the stomach, liver and intestine. It was identified from its morphological structure (cuticle with transverse striations, terminal mucron, larval tooth, filariform esophagus and blind intestine) (Figure 1).

Moreira et al. (2009), Takemoto et al. (2009), Bittencourt et al. (2014) and Tavares-Dias et al. (2014) reported that the presence of *Contracaecum* sp. may be mainly related to the geographical distribution of hosts, habitat and way of life, age and longevity, position in the trophic chain, volume of food ingested, ontogenetic changes in immunocompetence and diet and the likelihood of contact with infecting intermediate hosts in the environment.

C*ontracaecum* sp. is a parasite with zoonotic potential that has already been reported in several species of freshwater fish from Brazil (Benigno et al., 2012; Alcântara & Tavares-Dias, 2015; Rodrigues et al., 2017). The feeding habits of the fish evaluated in the present study (plankton, crustaceans, insects and seeds) (Leal et al., 2010; Froese & Pauly, 2016) predisposes them to occurrence of *Contracaecum* sp. According to Moreira et al. (2009), this parasite uses microcrustacean species as intermediate hosts and fish as secondary or paratenic intermediate hosts, while piscivorous birds are the definitive hosts.

In Brazil, the species *Contracaecum marginatum* uses the gastropod *Biomphalaria* spp. as the primary intermediate host and fish as a secondary intermediate host. The cycle is completed in piscivorous birds, which constitute the definitive hosts (Pinto & De Melo, 2013). Thus, in wild fish populations, such as those evaluated here, Alcântara & Tavares-Dias (2015) mentioned that parasite transmission occurred through ingestion of prey (intermediate hosts) and that the variability of the feeding behavior of predatory fish may have a strong influence on the distribution of parasite species.

Another point to be highlighted is the zoonotic potential of C*ontracaecum* sp. This has already been reported in several species of freshwater fish from Brazil (Benigno et al., 2012; Alcântara & Tavares-Dias, 2015; Rodrigues et al., 2017).

In the present study, larvae and adult forms of acanthocephalans were only found in the intestine of the host species *H. unitaeniatus*, at low prevalences (Table 1). In identifying these parasites, the size and shape of the body and proboscis, layout and number of hooks and spines on the proboscis, size of the pouch of the proboscis, shape of the wall and its number of layers, length of the lemnisci and shape of the cement gland were taken into consideration, as described by Eiras et al. (2006). Takemoto et al. (2009) conducted a survey of parasite species in 3,768 specimens of fish belonging to 72 species and identified low specificity of acanthocephalans, such that they affected seven different host species.

Histological analyses revealed liver alterations at stages I, II and III, both in *H. unitaeniatus* and in *C. bimaculatum* (Table 2). Due to the similarity of the alterations in the two species evaluated, which may have been related to the habits of the hosts and their food intake, we chose to present and discuss the results together.

**Table 2:** Frequency of liver lesions in *Hoplerythrinus unitaeniatus* and *Cichlasoma bimaculatum* originating from a quilombola community in Maranhão, Brazil.

**Stage**	**Histological changes to gills**	**N**	**Frequency**
**I**	Vacuolization	38	90.47
	Melanomacrophage center	33	78.57
	Deformation of the cell outline contour	28	66.67
	Nucleus at the periphery of the cell	22	52.38
	Cellular hypertrophy	3	7.14
	Nuclear hypertrophy	1	2.38
	Cellular atrophy	1	2.38
	Nuclear atrophy	1	2.38
**II**	Hyperemia	19	45.23
	Cytoplasmic degeneration	17	40.47
	Vacuolization nuclear	9	21.42
	Nuclear degeneration	6	14.28
	Cell disruption	5	11.90
**III**	Necrosis	4	9.52

Where: N= number of lesions.

**Figure 2:**
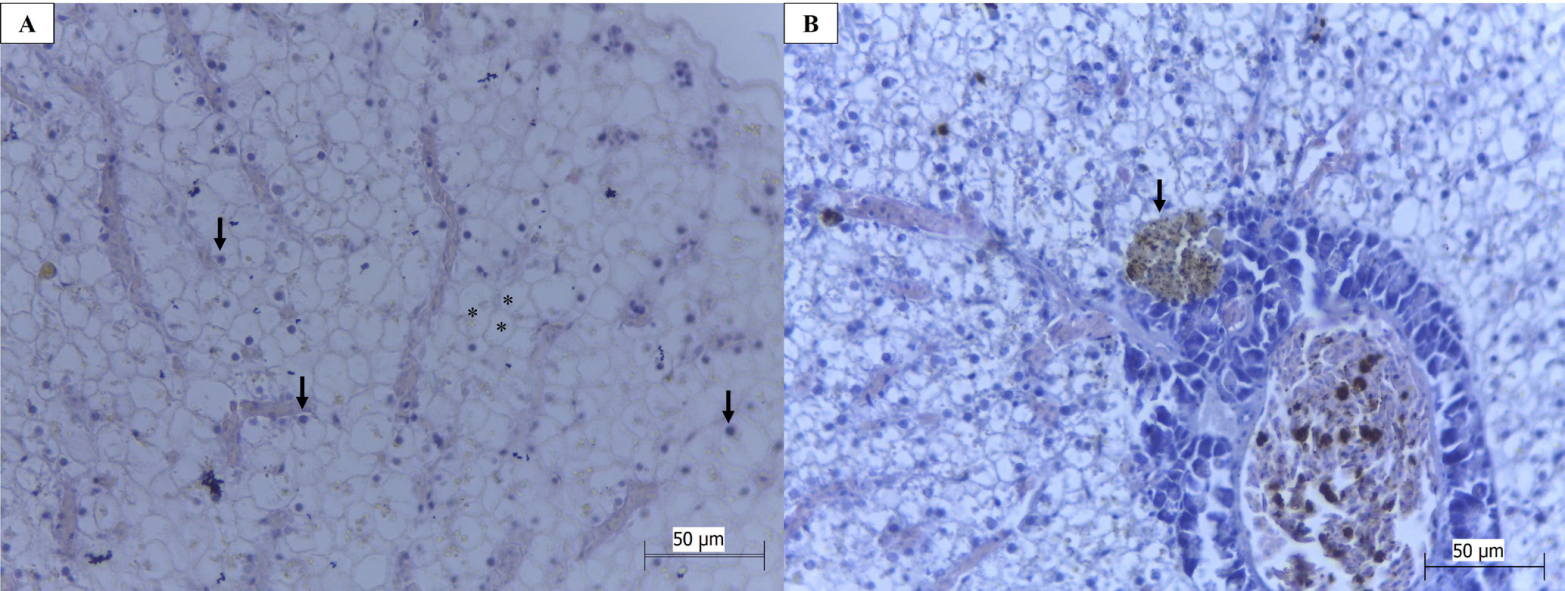
Photomicrographs of liver alterations *in Hoplerythrinus unitaeniatus* and *Cichlasoma bimaculatum* from a quilombola community in Maranhão, Brazil: (A) disorganization of the hepatic parenchyma through fat accumulation, presence of vacuoles inside the hepatocytes (*) and nuclei displaced to the periphery (arrows); (B) presence of melanomacrophage center (arrow). HE; objective lens 40X. **Source:** Authors' files. Bars 1A and 1B: = 50 μm.

Stage I lesions were seen frequently in the specimens evaluated, and they consisted mostly of vacuolization, nuclei in the periphery of the cells, deformation of the cell outline (Figure 2A) and melanomacrophage centers (Figure 2B).

Cytoplasmic vacuolization results from abnormal metabolization of lipids. It promotes displacement of the nucleus to the periphery of the cell, as well as degeneration of cytoplasm (Yancheva et al., 2016). The frequency of melanomacrophages in the liver is related to increased phagocytic activity as an immune response to lesions in individuals who are exposed to contaminants. It is characterized by accumulation of pigments inside cells, such as hemosiderin (Rabitto et al., 2005).

**Figure 3:**
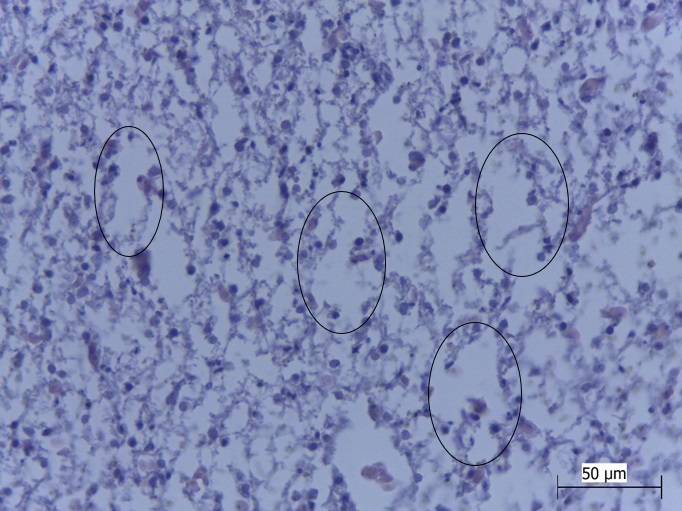
Photomicrograph of cell disruption and necrosis (circles) in the liver of *Cichlasoma bimaculatum* from a quilombola community, Maranhão, Brazil. HE; objective lens 40X. Bar: = 50 μm. **Source:** Authors' files.

Stage II lesions were also frequent in the specimens evaluated from both of the species that were examined and consisted mostly of nuclei in the periphery of cells (52.38%), hyperemia (45.23%), cytoplasmic degeneration (40.47%) and nuclear vacuolization (24.42%). Moreover, necrosis of the type seen in stage III lesions (Figure 3) was identified in 9.52% of the specimens evaluated. According to Rabitto et al. (2005), necrosis causes functional and structural damage to the liver of fish, decreases functionality and may cause organ failure. This consequently affects higher levels of biological organization.

**Figure 4:**
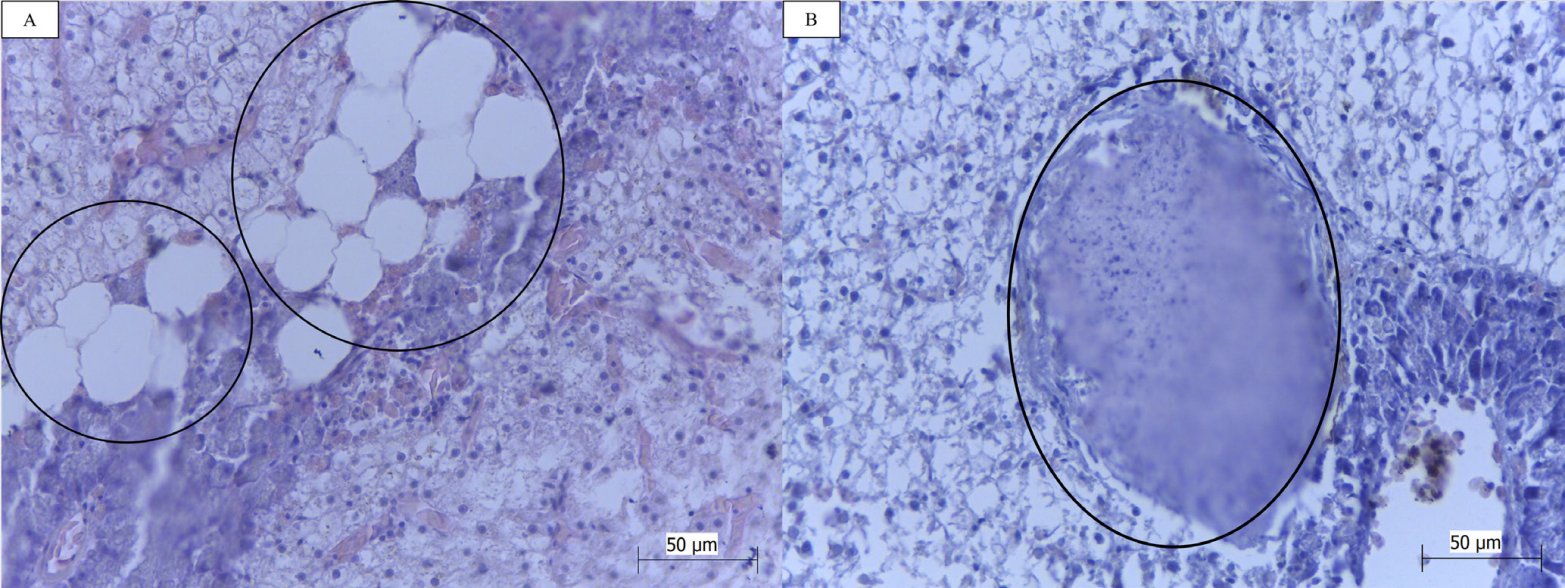
Photomicrographs of the liver of *Cichlasoma bimaculatum*, from a quilombola community in Maranhão, Brazil: (A) fatty degeneration (circles); (B) abscess (circle). HE; objective lens 40X. Bars 1A and 1B: = 50 μm. **Source:** Authors' files.

Fatty degeneration (n = 6/42; 14.29%; Figure 4A), inflammatory processes (n = 6/42; 14.29%), hemorrhage (n = 2/42; 4.76%); abscesses (n = 2/42; 4.76%; Figure 4B) and evident nucleoli (n = 2/421; 4.76%) were diagnosed in the present study but were not among the alterations described by Poleksic & Mitrovic-Tutundzic (1994).

The liver alterations that were identified in *H. unitaeniatus* and *C. bimaculatum* may have been related to the endoparasites that were found, considering that histological lesions were observed in all the parasitized hosts. According to Wester et al. (2002), histopathological studies are important tools for monitoring biological and ecological effects.

Histopathological evaluation of the liver may reveal a variety of types of lesion, such as inflammatory or degenerative alterations, necrosis, hyperplasia, fibrosis, vacuolization, foci of cell alteration, neoplasia, etc. (Lang et al., 2006). Velasco et al. (2012) evaluated the morphological characteristics of a hepatopancreatic coccid and observed that oocysts promoted slight compression of the hepatocytes adjacent to the locations of the parasite.

Through calculating the histological alteration index (HAI), it was found that 26.19% (n = 11/42) of the specimens presented normal tissue functioning; 26.19% (n = 11/42) had mild to moderate tissue damage; 38.09% (n = 16/42) had moderate to severe tissue modification with the presence of endoparasites; and 9.52% (n = 4/42) had irreparable tissue damage with findings of larvae of *Contracaecum* sp. encysted in the organ.

## Conclusions

This was the first study in the state of Maranhão on endoparasite diversity in the native fish species *H. unitaeniatus* and *C. bimaculatum*. High prevalence of infection of both of these species with *Contracaecum* sp. was observed. Only in the host species *H. unitaeniatus* were adults and larvae of acanthocephalans identified. It can be concluded that the histological liver lesions identified in our study may be adaptive responses by the fish to parasitic infection.

## References

[B001] Alcântara NM, Tavares-Dias M (2015). Structure of the parasites communities in two Erythrinidae fish from Amazon river system (Brazil). Rev Bras Parasitol Vet.

[B002] Alves FL, Maruo VM, Mazzinghy CL (2019). Fauna parasitária de peixes da família Pimelodidae. Rev Cien Med Vet.

[B003] Benigno RN, Clemente SC, Matos ER, Pinto RM, Gomes DC, Knoff M (2012). Nematodes in *Hoplerytrinus unita*eniatus, *Hoplias malabaricus* and *Pygocentrus nattereri* (pisces characiformes) in Marajó Island, Brazil. Rev Bras Parasitol Vet.

[B004] Benigno RNM, Knoff M, Matos ER, Gomes DC, Pinto RM, São Clemente SC (2014). An Acad Bras Cienc.

[B005] Bittencourt LS, Pinheiro DA, Cárdenas MQ, Fernandes BM, Tavares-Dias M (2014). Parasites of native Cichlidae populations and invasive *Oreochromis niloticus* (Linnaeus, 1758) in tributary of Amazonas River (Brazil). Rev Bras Parasitol Vet.

[B006] Bush AO, Lafferty KD, Lotz JM, Shostak AW (1997). Parasitology meets ecology on its own terms: Margolis et al. revisited. J Parasitol.

[B007] Caputo LFG, Gitirana LB, Manso PPA, Molinaro EM, Caputo LFG, Amendoeira MRR (2010). Conceitos e métodos para formação de profissionais em laboratório de saúde.

[B008] Eiras JC, Takemoto RM, Pavanelli GC (2006). Métodos de estudo e técnicas laboratoriais em parasitologia de peixes..

[B009] Froese R, Pauly D (2016). FishBase.

[B010] Fujimoto RY, Hide DM, Paixão PEG, Abe HA, Dias JAR, Sousa NC (2019). Fauna parasitária e relação parasito-hospedeiro de tambaquis criados na região do Baixo São Francisco, nordeste do Brasil. Arq Bras Med Vet Zootec.

[B011] Jaramillo-Colorado BE, Arroyo-Salgado B, Ruiz-Garcés LC (2015). Organochlorine pesticides and parasites in *Mugil incilis* collected in Cartagena Bay, Colombia. Environ Sci Pollut Res Int.

[B012] Jerônimo GT, Tavares-Dias M, Martins ML, Ishikawa MM (2012). Manual para coleta de parasitos em peixes de cultivo..

[B013] Koeppen W (1948). Climatologia: com um estudio de los clima de la tierra..

[B014] Lang T, Wosniok W, Barsiene J, Broeg K, Kopecka JE, Parkkonen J (2006). Liver histopathology in Baltic flounder (*Platichthys flesus*) as indicator of biological effects of contaminants. Mar Pollut Bull.

[B015] Leal ME, Klein GF, Schulz UH, Albornoz PL (2010). Primeiro registro e aspectos ecológicos de *Hoplerythrinus unitaeniatus* (Agassiz, 1829) (Characiformes, Erythrinidae) como espécie introduzida na Bacia do Rio dos Sinos, RS, Brasil. Biota Neotrop.

[B016] Martins ML, Onaka EM, Fenerick J (2005). Larval *Contracaecum* sp. (Nematoda: Anisakidae) in *Hoplias malabaricus* and *Hoplerythrinus unitaeniatus* (Osteichthyes: Erythrinidae) of economic importance in occidental marshlands of Maranhão, Brazil. Vet Parasitol.

[B017] Moravec F, Ogawa K, Suziki M, Miyazaki K, Donai H (2002). On two species of *Philometra* (Nematoda, Philometridae) from the serranid fish *Epinephelus septemfasciatus* in Japan. Acta Parasitol.

[B018] Moreira LHA, Takemoto RM, Yamada FH, Ceschini TL, Pavanelli GC (2009). Ecological aspects of metazoan endoparasites of *Metynnis lippincottianus* (Cope, 1870) (Characidae) from Upper Paraná River floodplain, Brazil. Helminthol.

[B019] Pardo SC, Zumaque MA, Noble CH, Suarez MH (2008). *Contracaecum* sp. (Anisakidae) en el pez *Hoplias malabaricus*, capturado en la ciénaga grande de lorica, Córdoba. Rev Mvz Cordoba.

[B020] Pavanelli GC, Eiras JC, Takemoto RM (2008). Doenças de peixes: profilaxia, diagnóstico e tratamento..

[B021] Pereira DP, Santos DMS, Carvalho AV, Cruz CF, Carvalho Neta RNF (2014). Alterações morfológicas em brânquias de *Oreochromis niloticus (*Pisces, Cichlidae) como biomarcadores de poluição aquática na Laguna da Jansen, São Luís, MA (Brasil). Biosci J.

[B022] Pereira PRM, Rodrigues TCS, Viegas JC (2016). Diagnóstico ambiental e caracterização morfométrica das Microbacias Hidrográficas de Pedro do Rosário, Amazônia Maranhense (Brasil). Rev Bras Gest Ambient Sustentabilidade.

[B023] Pinto HA, De Melo AL (2013). A checklist of cercariae (Trematoda: Digenea) in molluscs from Brazil. Zootaxa.

[B024] Poleksic V, Mitrovic-Tutundzic V (1994). Fish gills as a monitor of sublethal and chronic effects of pollution..

[B025] Rabitto IS, Alves Costa JRM, Silva de Assis HC, Pelletier EE, Akaishi FM, Anjos A (2005). Effects of dietary Pb (II) and tributyltin on neotropial fish, *Hoplias malabaricus*: histopathological and biochemical findings. Ecotoxicol Environ Saf.

[B026] Rocha RM, Coelho RP, Montes CS, Santos SSD, Ferreira MAP (2010). Avaliação histopatológica do fígado de *Brachyplatystoma rousseauxii* (Castelnau, 1855) da Baía do Guajará, Belém, Pará. Cienc Anim Bras.

[B027] Rodrigues LC, Santos ACG, Ferreira EM, Teófilo TS, Pereira DM, Costa FN (2017). Aspectos parasitológicos da traíra (*Hoplias malabaricus*) proveniente da cidade de São Bento, MA. Arq Bras Med Vet Zootec.

[B028] Takemoto RM, Pavanelli GC, Lizama MAP, Lacerda ACF, Yamada FH, Moreira LHA (2009). Diversity of parasites of fish from the Upper Paraná River floodplain, Brazil. Braz J Biol.

[B029] Tavares-Dias M, Alves Gonçalves R, Brito Oliveira MS, Rigôr Neves L (2017). Ecological aspects of the parasites in *Cichlasoma bimaculatum* (Cichlidae), ornamental fish from the Brazilian Amazon. Acta Biol Colomb.

[B030] Tavares-Dias M, Oliveira MSB, Gonçalves RA, Silva LMA (2014). Ecology and seasonal variation of parasites in wild *Aequidens tetramerus*, a Cichlidae from the Amazon. Acta Parasitol.

[B031] Velasco M, Videira M, Matos P, São Clemente SC, Sanches O, Matos E (2012). Morfologia e nova ocorrência de um coccídio hepatopancreático parasita de peixe amazônico. Rev Cienc Agrar.

[B032] Wester PW, Van Der Ven LTM, Vethaak AD, Grinwis GCM, Vos JG (2002). Aquatic toxicology: opportunities for enhancement through histopathology. Environ Toxicol Pharmacol.

[B033] Yancheva V, Velcheva I, Stoyanova S, Georgieva E (2016). Histological biomarkers in fish as a tool in ecological risk assessment and monitoring programs: a review. Appl Ecol Environ Res.

